# Cost-effectiveness of measles and rubella elimination in low-income and middle-income countries

**DOI:** 10.1136/bmjgh-2022-011526

**Published:** 2023-07-10

**Authors:** Ann Levin, Colleen Burgess, Stephanie Shendale, Winthrop Morgan, Raymond Cw Hutubessy, Measles–rubella Eradication Modelling Group, Amy Winter, Mark Jit

**Affiliations:** 1 Levin & Morgan LLC, Bethesda, Maryland, USA; 2 Self-employed, Phoenix, Arizona, USA; 3 World Health Organization, Geneva, Switzerland; 4 Faculty of Public Health and Policy, London School of Hygiene and Tropical Medicine, London, UK

**Keywords:** immunisation, measles, health economics

## Abstract

**Background:**

Since 2000, the incidence of measles and rubella has declined as measles–rubella (MR) vaccine coverage increased due to intensified routine immunisation (RI) and supplementary immunisation activities (SIAs). The World Health Assembly commissioned a feasibility assessment of eliminating measles and rubella. The objective of this paper is to present the findings of cost-effectiveness analysis (CEA) of ramping up MR vaccination with a goal of eliminating transmission in every country.

**Methods:**

We used projections of impact of routine and SIAs during 2018–2047 for four scenarios of ramping up MR vaccination. These were combined with economic parameters to estimate costs and disability-adjusted life years averted under each scenario. Data from the literature were used for estimating the cost of increasing routine coverage, timing of SIAs and introduction of rubella vaccine in countries.

**Results:**

The CEA showed that all three scenarios with ramping up coverage above the current trend were more cost-effective in most countries than the 2018 trend for both measles and rubella. When the measles and rubella scenarios were compared with each other, the most cost-effective scenario was likely to be the most accelerated one. Even though this scenario is costlier, it averts more cases and deaths and substantially reduces the cost of treatment.

**Conclusions:**

The Intensified Investment scenario is likely the most cost-effective of the vaccination scenarios evaluated for reaching both measles and rubella disease elimination. Some data gaps on costs of increasing coverage were identified and future efforts should focus on filling these gaps.

**WHAT IS ALREADY KNOWN ON THIS TOPIC**

- Previous studies have found that measles elimination is likely to be more cost-effective than measles mortality reduction strategies.

**WHAT THIS STUDY ADDS**

- This study revealed that, compared with other measles–rubella vaccination strategies to reach elimination, a more accelerated ramp-up strategy is likely to be the most cost-effective. This study differs from previous studies since it includes rubella vaccination and uses multiple dynamic models for comparison.

**HOW THIS STUDY MIGHT AFFECT RESEARCH, PRACTICE OR POLICY**

- This study provides additional evidence to policymakers that accelerating measles–rubella vaccination with an objective of eliminating the diseases is a good investment. In addition, future research on the costs of increasing vaccination will further inform these estimates.

## Background/introduction

 Measles is one of the most infectious childhood diseases. It also causes a substantial economic burden on households in low-income and middle-income countries. The societal cost of treating a measles case, including lost productivity of caregivers, ranges from 2018 US$20 and US$104 in Uganda,^1^ to 2018 US$18 and US$159 in Bangladesh^2^ for ambulatory and hospitalised cases, respectively, and finally 2018 $802 per hospitalised case in Fujian, China.^3^

Rubella infections are milder than those of measles. However, when pregnant women are infected with rubella early in pregnancy, they often give birth to infants with congenital rubella syndrome (CRS), a condition that can cause severe disabilities such as hearing impairments, eye and heart defects, autism, diabetes and thyroid dysfunction. As a result, the economic burden is very steep—ranging from annual costs of 2012 US$4261 per CRS case (outbreak in 2004) in Brazil,^4^ to 2013 US$6455 per CRS case (outbreak in 2011) in Romania.^5^ Lifetime costs are particularly high for CRS cases at 2004 US$61 284 in Brazil and 2013 US$44 051 in Romania.

Two doses of a measles and rubella-containing vaccine give a high level of protection against both viruses. Progress has been made against measles and rubella infections as measles–rubella (MR) vaccine coverage was increased through intensified routine immunisation (RI) and supplementary immunisation activities (SIAs) of children aged <5 years. Between 2000 and 2019, estimated measles deaths declined 73%^6^ and reported rubella infections declined 98% from 671 000 to 15 000 cases.^7^ Most of the decline in the number of reported infections occurred between 2000 and 2010 since vaccination coverage rates stagnated in the 2010s and even declined recently during the COVID-19 pandemic. Today, there are still many cases and deaths from measles worldwide—9.8 million cases and 207 500 deaths in 2019^8^—as well as cases of CRS.

The WHO World Health Assembly (WHA) called for a feasibility assessment of measles and rubella eradication.^9^ Following the call, the WHO Strategic Advisory Group of Experts on Immunization (SAGE) requested that the SAGE MR working group and Immunization and Vaccines Related Implementation Research Advisory Committee (IVIR-AC) review MR eradication modelling efforts in August 2018.^10^ In March 2019, they commissioned both health and economic modelling to inform the feasibility of eliminating these diseases in each country and cost-effectiveness analysis (CEA) of various service delivery scenarios to eliminate both diseases. The study was funded by WHO, Gavi, the Vaccine Alliance, US Centers for Disease Control and Prevention and the Bill & Melinda Gates Foundation.

The objective of this paper is to present the findings of the CEA of ramping up MR vaccination over the period 2018–2047, toward a goal of global disease elimination.

## Methods

To estimate cost-effectiveness of MR vaccination, the impact of four different vaccination scenarios on measles and rubella incidence and mortality for 93 low-income and middle-income countries was calculated for the years 2018–2047, using mathematical models of measles and rubella transmission.^11^ It was assumed that the countries would remain in their 2018 World Bank income group^12^ during the period. The four scenarios were: (1) Base Case, where routine vaccination and SIA coverage of children aged <5 years remained at 2018 levels; (2) Continuing Trends, where limited annual improvements based on historic trends in coverage were assumed; (3) Constant Improvement, where coverage rates had linear annual increases of one percentage point and were assumed to ramp up faster than historic trends in Gavi, the Vaccine Alliance-supported countries; and (4) Intensified Investment, where coverage had compounding increases of 4.4% annually for all countries and SIAs were conducted more frequently. Additional details on the scenarios employed in the analysis, and the list of countries included, are provided in the online supplemental material.

Outcomes from the transmission models were then combined with economic parameters to estimate the impact of each vaccination scenario on health (disability) and cost outcomes. The health outcomes and costs for measles vaccination and for rubella vaccination were calculated separately due to their different efficacy, even though these are delivered via a combined MR vaccine. In addition, although all have measles-containing vaccination in their immunisation programmes, some countries still deliver single antigen measles-containing vaccine (without rubella). Furthermore, the outcomes of MR vaccination are measured differently. Vaccine coverage, cost of vaccination and treatment are assumed to vary by the scenario and country income group (World Bank lower, lower-middle and upper-middle income groupings in 2018 US$). The vaccine coverage scenario assumptions were based on expert discussions and projections made by US Centers for Disease Control and Prevention (see online supplemental material and Winter *et al*^11^ for details).

To estimate incremental cost-effectiveness, we compared the incremental costs and incremental disability-adjusted life years (DALYs) incurred for each scenario to the next most costly scenario. Incremental costs consisted of the cost of the vaccination programme itself minus the cost of treating measles cases or CRS cases (no other rubella treatment costs were modelled) which were averted. Treatment costs and DALYs incurred in each scenario were estimated from epidemiological model projections of the impact of vaccination (lives saved and non-fatal measles cases and CRS cases averted). If a scenario incurred both negative incremental costs and negative incremental DALYs compared with its comparator, then it was deemed to dominate that scenario. Otherwise, cost-effectiveness was determined by comparing the incremental cost-effectiveness ratio against a societal willingness-to-pay threshold. The willingness-to-pay thresholds used in this study are from Ochalek.^13^ These thresholds vary by country and account for health opportunity costs. Each country’s incremental cost-effectiveness ratios were compared with Ochalek’s DALY 3 estimate for the measles analysis. The threshold for a neighbouring country was used when none was available for a country. In the case of rubella vaccine, the global ICERs were used instead of the country ICERs due to the low number of CRS in smaller countries.

A healthcare provider perspective was used. Costs were discounted at 3%, while DALYs were discounted at either 3% or 0% following WHO guidelines.^14^ All costs were inflated to constant 2018 US$ values. The time horizon for the analysis was 30 years, from 2018 to 2047.

### Modelling health outcomes

The measles and rubella transmission models were used to project the number of measles and CRS cases, deaths and DALYs averted for the four different scenarios of MR vaccination, employing demographic data provided by the UN population projections and on historical MR vaccination coverage provided by WHO (full details on the transmission models are provided in Winter *et al*^11^). Two models were used for each disease; here we show results from the Penn State University model for measles and the Johns Hopkins University model for rubella. Results from the DynaMICE model for measles and Public Health England model for rubella are shown in the online supplemental material; these give qualitatively similar results and produce similar incremental cost-effectiveness ratios for the various scenarios.

All four models estimated the impact of different vaccine scenarios on cases, deaths and DALYs incurred using dynamic transmission compartmental SIR-type models (ie, with compartments for susceptible, infected and recovered people). In addition, they had compartments for vaccinated people and children protected through maternal-derived immunity. Additional details of the transmission models are provided in Winters *et al*.^11^

### Estimating cost outcomes

#### Routine vaccination costs

We estimated separate costs for vaccine delivery via the RI programme and SIAs.

For routine delivery, we searched the ICAN immunisation cost catalogue^15^ for peer-reviewed and grey literature articles on the costs of MR vaccination delivered at RI sites, defined for this study as fixed facilities with weekly or daily sessions, including outreach delivery sites. Studies on delivery costs were classified according to whether they used average or incremental costing and by World Bank country income group (low, lower-middle and upper-middle). The cost estimates of delivering a dose of measles or rubella-containing vaccine were expected to differ by country income group due to variation in labour, commodity, and supply costs.

Six studies of RI that estimated incremental costs were identified.^16^,^21^ Three of these studies were part of the Gates Foundation-funded EPIC (Expanded Program on Immunization Costing and Financing of Routine Immunization) studies, while the other three were based on non-EPIC studies.

The mean delivery costs were estimated separately depending on whether countries were projected to be using single-antigen measles or MR vaccination. The assumptions made on costs of MR vaccination are shown in online supplemental table 2. The vaccine price for MR vaccine was taken from UNICEF Supply Division prices for low-income countries,^22^ and from PAHO Revolving Fund prices^23^ for lower-middle and upper-middle income countries, respectively.

The incremental cost per dose delivered is expected to increase (although not necessarily linearly) with coverage levels because of the additional costs required to reach hard-to-reach populations. Some activities that are assumed to intensify with increasing coverage are communications/social mobilisation, cold chain expansion, and surveillance.

The team used results from a systematic review of studies on the cost of increasing coverage by Ozawa *et al*^24^ to estimate the elasticity of vaccine delivery costs to coverage. This review summarised 56 studies on delivery costs at various levels of coverage of one or more vaccine-preventable diseases. In our analysis, studies were removed if they estimated SIA costs rather than RI, or focused on vaccine introduction since these incorporate start-up costs that do not reflect the cost of maintaining existing programmes. Eight studies met these criteria. Studies from high-income countries were analysed separately from those in low-income and middle-income countries to provide information on the rate of increase in costs as coverage increases. In addition, we reviewed studies on costs of surveillance^25^,^27^ so that we could adjust the total for these. We added US$0.05 per dose when increasing from low coverage to medium coverage (60%–79%), US$0.10 when increasing from 80%–89% and US$0.20 for 90%+. Studies in low-income and lower-middle income countries have initial coverage around 50%–60%, while the studies in high-income countries have coverage over a wider range around 40%–80%. Based on these data, the cost of increasing vaccination coverage was estimated by current coverage and income levels, as can be seen in table 1.

**Table 1:** Discounted measles vaccination costs, cases, treatment costs, net costs, DALYS averted and cost per DALY averted in 2018 US$ over 2018–2047, 93 LMICs

Discounted measles vaccination costs, treatment costs, net costs and DALYs averted					
Scenario	Discounted*vaccination costs(millions)	Cases (millions)	Measles treatment costs(millions)	DALYs(millions)	Total costs(millions)
Base Case	US$10 459	490.4	US$10 014	783	US$20 473
Continuing Trends	US$11 918	213.8	US$6158	332	US$18 076
Constant Improvement	US$13 857	158.0	US$4724	230	US$18 580
Intensified Investment	US$14 794	111.5	US$3537	158	US$18 332

Cost per DALY averted					
Scenario	Comparator	Net costs (millions)	DALYs averted(millions)	Cost per DALY averted	Percentage of countries meeting Ochalek threshold
Continuing Trends	Base Case	−US$2397	451	Dominant	89
Constant Improvement	Continuing Trends	US$505	102	US$4.94	76
Intensified Investment	Continuing Trends	US$256	174	US$1.47	70
Intensified Investment	Constant Improvement	−US$249	72	Dominant	80

* The costs were discounted with a discount rate of 3%. Note: The cost-effectiveness threshold for the 92 LMICs, were estimated based on cost per DALYs averted that reflect health opportunity costs on mortality of changes in health expenditures.^13^

DALYs, disability-adjusted life years ; LMICs, low-income and middle-income countries.

The authors also conducted literature reviews on the delivery costs of SIAs and treatment costs. We found two articles on SIA costs: (1) a systematic review of SIA costs by Gandhi *et al*,^28^ and (2) a six-country study of cost-effectiveness of alternative measles strategies to reach measles eradication by Levin *et al*.^29^ We also found a small number of studies on treatment costs for measles and CRS conducted in Latin America,^30^ Ethiopia,^31^ and^32^ Bangladesh, Tajikistan and Uganda,^29^ and in Romania.^5^ The bottom half of table 1 shows the direct treatment costs used in the estimation of the scenarios that were adjusted to 2018 US$ using the US Consumer Price Index.

### Sensitivity analyses for model parameters

Two types of sensitivity analyses were conducted: one-way direct sensitivity analysis (DSA) and probabilistic sensitivity analysis (PSA). The DSA was performed on cost parameters, varying vaccine price (cost per dose for routine and campaign vaccination) and cost for treatment, stratified by country income group. These nine cost parameters were varied across ±20%; as an additional sensitivity analysis, routine costs per dose were varied up to +100%. The discounting factor for DALYs was also varied as either 3% (baseline assumption) or 0%. The impact of cost parameter variation was measured as change in net present value of the global costs, calculated over the time period from 2018 to 2047. Measles and CRS case-fatality risks (CFRs) were also included in the sensitivity analysis. Since health outcomes were provided by the modelling groups, we applied a multiplicative factor to the number of DALYs for each scenario, as a surrogate for CFR, with a variation of±20%.

PSA was conducted to analyse the sensitivity of the results to simultaneous changes in multiple parameters. The PSA was performed on the same set of parameters (excluding the discounting rate), randomly selecting values from uniform distributions for each parameter simultaneously and evaluating the effect of these variations on global discounted costs. Two hundred sets of parameter value variations were considered, with samples drawn from a range of ±20% around baseline values via Latin Hypercube Sampling.^32 33^

### Patient and public involvement

Patients were not included in this modelling study.

## Results

### Measles elimination cost-effectiveness

Based on the assumptions made, the annual total measles cases can reach up to 40 million during the peak of multiyear cycles for the duration of the simulation period under the Base Case scenario. Under the Intensified Investment scenario, total annual case numbers rapidly decrease with higher vaccine coverage. Thus, while vaccination costs for Intensified Investment are higher than those for Base Case, this is offset by substantially lower treatment costs resulting from decreased caseloads.

**Figure 1:**
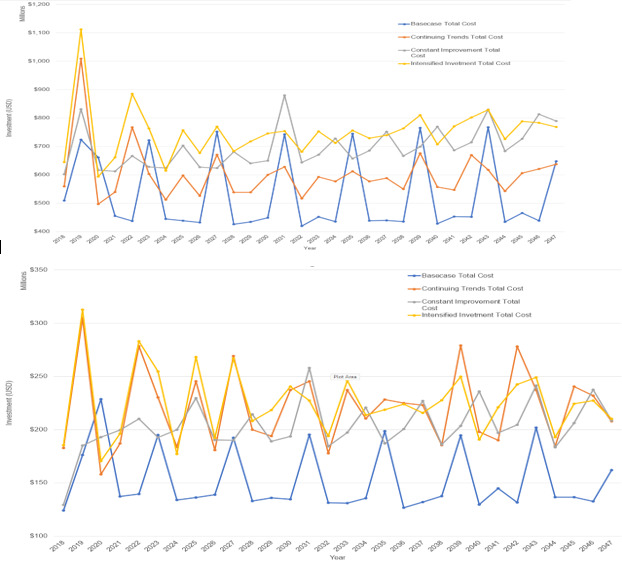
Vaccination cost by scenario, 2018–2047 (2018 US$), 93 low-income and middle-income countries for (**A**) measles, and (**B**) rubella.

Figure 1A,B shows the MR vaccination costs by scenario over the time period of 2018–2047 (in constant 2018 US$). The variation in annual costs occurs since SIAs occur every 3–5 years in many countries.

Table 1 shows the discounted measles vaccination costs, treatment costs and DALYs averted for each scenario over a time period of 2018–2047. While vaccination costs are highest for the Intensified Investment scenario, net costs are highest for the Base Case due to the high cost of measles treatment. The other scenarios have fewer cases and DALYs and treatment costs.

The second part of table 1 shows the cost per DALY averted for the measles scenarios compared with the next most expensive non-dominated alternative. The Continuing Trends scenario is more cost-effective than the Base Case in 89% of the countries since costs are lower and DALYs averted are greater, and thus is dominant. When the Constant Improvement scenario is compared with Continuing Trends, the former has slightly greater net costs but averts more DALYs. Finally, when the Intensified Investment scenario is compared with Continuing Trends, the Intensified Investment scenario again costs more but has a very low incremental cost-effectiveness ratio. In addition, it dominates the Constant Improvement scenario in 80% of the countries. Thus, the Intensified Investment scenario is estimated likely to be the most cost-effective of all scenarios for the majority of countries when using the baseline values.

Table 2 shows discounted rubella vaccination costs, CRS cases, CRS treatment costs, DALYs, cost per DALY averted and whether the Ochalek threshold is met. The costs are highest for the Base Case because of the high costs of treating CRS and lowest for the Constant Improvement scenario.

**Table 2:** Discounted rubella vaccination costs, CRS cases, treatment costs, net Costs, DALYs averted and cost per DALY averted in 2018 US$ over 2018–2047, 93 LMICs

Discounted rubella vaccination costs, treatment costs, net costs and DALYs averted					
Scenario	Discounted*vaccination costs(millions)	CRS cases (000 s)	CRStreatment costs(millions)	DALYs(millions)	Total costs(millions)
Base Case	US$2908	892	US$3980	23.8	US$6887
Continuing Trends	US$4473	252	US$1457	6.6	US$5931
Constant Improvement	US$4113	298	US$1746	7.8	US$5859
Intensified Investment	US$4611	223	US$1296	5.9	US$5907

Cost per DALY averted				
Scenario	Comparator	Net Costs (millions)	DALYs averted(millions)	Cost per DALY averted
Continuing Trends	Base Case	−US$420.8	9.8	Dominant
Constant Improvement	Continuing Trends	US$6.4	1.2	US$5.3
Intensified Investment	Continuing Trends	US$47.6	1.6	US$29.3
Intensified Investment	Constant Improvement	US$41.1	0.4	US$101.3

* The costs were discounted with a discount rate of 3%. Note: the cost-effectiveness threshold for the 92 LMICs, were estimated based on cost per DALYs averted that reflect health opportunity costs on mortality of changes in health expenditures.^13^

DALYs, disability-adjusted life years; LMICs, low-income and middle-income countries.

The cost per DALY averted for the rubella scenarios compared with one another is shown in the bottom half of table 2. The results indicate that the Continuing Trends scenario dominates the Base Case scenario, and Constant Improvement is more cost-effective than Continuing Trends. Further, the Intensified Investment scenario dominates the Continuing Trends scenario. Finally, the Intensified Investment scenario can be considered to be more cost-effective than the Constant Improvement. Thus, it can be concluded that the Intensified Investment scenario is the most cost-effective one, as compared with the other scenarios.

#### Sensitivity analysis

**Figure 2:**
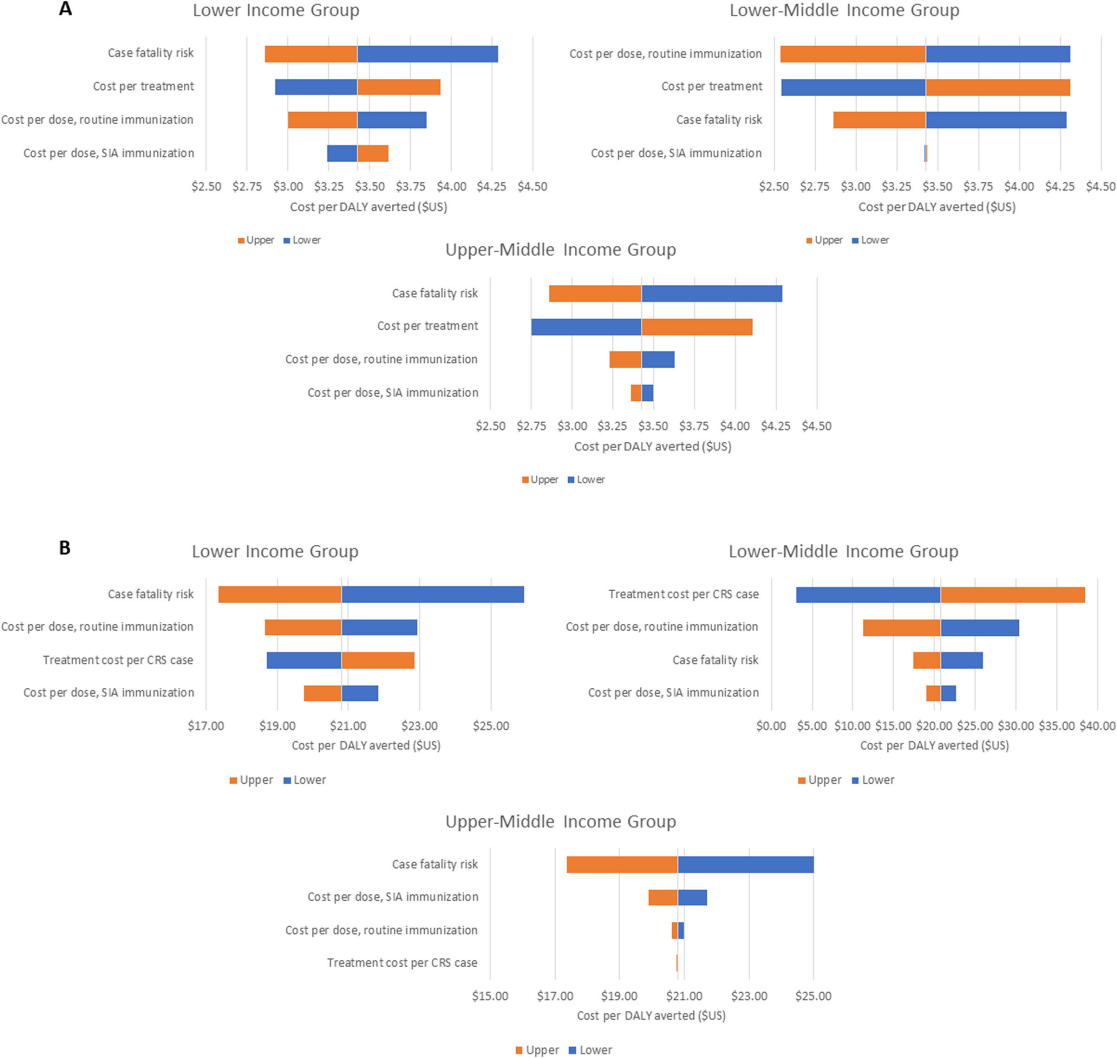
Univariate sensitivity analysis of incremental cost-effectiveness ratio for intensified investment scenario versus base case by country income group, 2018 US$, for (**A**) measles and (**B**) rubella. CRS, congenital rubella syndrome; DALYs, disability-adjusted life years; SIAs, supplementary immunisation activities.

Figure 2A shows tornado graphs of the one-way sensitivity analyses (DSA) for measles for the cost-effectiveness of the Intensified Investment scenario compared with the Base Case when parameters are varied by ±20%, stratified by country income group. Note that the cost per dose of a RI includes both the procurement and delivery cost. The DSA shows that the cost per DALY averted is most sensitive to changes in the measles case-fatality risk for low-income and upper-middle income groups—that is, as CFR increases, the number of DALYs averted with measles vaccination increases, leading to a decrease in the cost per DALY averted. For the lower-middle income group, the cost per DALY averted is more sensitive to changes in two parameters—the cost of a RI dose and treatment cost. The sensitivity to the RI cost per dose may be because increases in RI costs are associated with increases in coverage which lead to reductions in the number of cases. That is, as the vaccine price increases and coverage is ramped up, vaccination costs increase but total costs decline due to lower treatment costs occurring with more vaccination—and a lower cost per DALY averted. Cost per DALY averted in lower-middle income countries is likely to be more sensitive to the routine vaccination price since these countries would be having fewer campaigns than in lower income countries. The sensitivity to the treatment cost is because total costs will increase (or decrease) as treatment costs are augmented. For upper middle-income countries, treatment costs are higher than in lower-middle income countries and have more of an impact on cost per DALY averted than the cost of a RI dose.

The cost per DALY averted is less sensitive to changes in the cost per SIA vaccine dose delivered. The lower sensitivity of the cost per DALY averted to changes in the price per SIA vaccine implies that case treatment and disease-related deaths are a greater driver of the cost-effectiveness of a vaccination strategy than are the costs of campaign vaccinations.

When the cost per routine dose is varied by +100% (not shown in figure 2), most of the cost-effectiveness results are similar to when the variation was 20%, although not as cost-saving. At the highest routine cost per dose (+100%), the Increasing Investment strategy is cost saving compared with the Base Case for lower and upper-middle income countries, but less cost saving than at the+20% range. For lower-middle income countries, at the highest routine cost per dose (+100%) the Increasing Investment strategy costs more than the Base Case strategy and is below the willingness-to-pay threshold, although not cost-saving.

Figure 2B shows that the cost per DALY averted for rubella vaccination is most sensitive to the case-fatality risk for the low-income and upper-middle income country groups, while it is most sensitive to the treatment cost per CRS case for the lower-middle income country group. As in the case of measles, with increases in the case-fatality risk, the number of DALYs averted with rubella vaccination increases, leading to a decrease in the cost per DALY averted. For the lower-middle income country group, the cost per DALY averted is sensitive to the treatment cost per CRS case, due to the high cost of treatment relative to the cost of vaccination in this income group. The sensitivity of the treatment cost per CRS case is greater for the lower-middle income country group since the differential in cost is greater than for the low-income or upper-middle-income countries.

**Figure 3:**
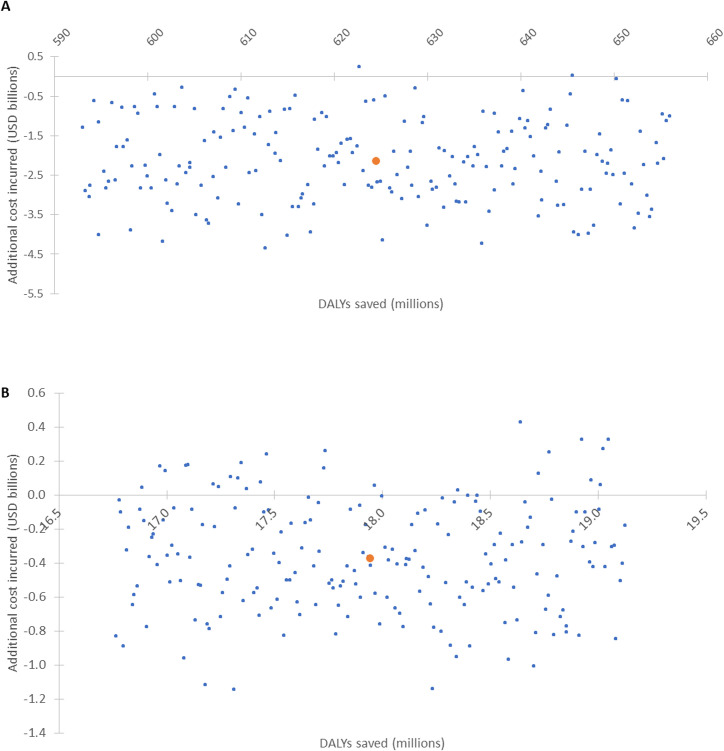
Probabilistic sensitivity analysis of intensified investment scenario compared to Base Case, 2018 US$, for (**A**) measles and (**B**) rubella. DALYs, disability-adjusted life years

Figure 3A,B shows the results of the measles and rubella PSAs, respectively, comparing the Intensified Investment scenario to the Base Case. Intensified Investment has more DALYs averted and lower costs in all but a few parameter iterations in comparison to the Base Case for measles—and for the majority (88%) of parameter iterations for rubella—indicating that it is more cost-effective even with some degree of variation in parameters. The other two scenarios were also found to be more cost-effective than the Base Case although the Intensified Investment scenario is the most cost-effective compared with the other scenarios.

## Discussion

Our analysis shows that greater effort in improving MR vaccine coverage towards elimination goals is a good investment economically. The CEA showed that all three scenarios involving higher vaccine coverage were more cost-effective than the Base Case scenario that assumes 2018 levels of routine and SIA vaccination continue, for both measles and rubella, based on the assumptions made in the analyses.

In these analyses, our estimate of vaccination costs does not include the funding for additional activities required to reach elimination. Specifically, additional activities required to reach global elimination include intensified surveillance, timely responses to outbreaks, and social mobilisation and communications. We did not estimate these additional costs since the modellers did not project the frequency of outbreaks that are likely to occur. In addition, there are limited data on the rate of increase in these costs to reach elimination. We also did not examine the economic benefits of achieving measles and/or rubella global eradication, which would provide a stream of benefits that would last indefinitely without requiring much investment in vaccination once eradication is achieved.

For measles, discounted estimated vaccination costs (without costs for additional activities to reach global elimination) across the 93 low-income and middle-income countries modelled during 2018–2047 range from US$10.5 billion for the Base Case to US$14.8 billion for Intensified Investment. In addition, measles treatment costs are assumed to decline from US$10.0 billion for the Base Case to US$3.5 billion for Intensified Investment as cases decline with additional vaccination. As cases decline, the DALYs also decrease from 783 million in the Base Case to 158 million in the Intensified Investment scenario. When the measles scenarios were compared with each other, the most cost-effective scenario was Intensified Investment.

For rubella, the discounted estimated vaccination costs (without costs of additional activities to achieve global elimination) for the 93 low-income and middle-income countries modelled during 2018–2047 range from US$2.9 billion for the Base Case to US$4.6 billion for the Intensified Investment scenario. With the decline in CRS cases, the DALYs also decrease from 23.8 million in the Base Case to 5.9 million in the Intensified Investment scenario. Similar to measles, the Intensified Investment scenario for rubella is the most cost-effective when compared with the other scenarios since it averted more DALYs and had lower net costs (or slightly higher costs when compared with Constant Improvement).

In addition to being the most cost-effective, the Intensified Investment scenario is also the most likely to produce reductions in cases and deaths in countries that would achieve the threshold for elimination of measles and rubella, based on the results of the modelling.^11^ For measles, our previous modelling analysis found that the threshold of less than 5 cases per 1 000 000 is more likely to be reached in countries that are projected to reach high levels of coverage. In addition, the analysis found that reaching the elimination threshold is more likely to be achieved for rubella than measles due to the differing characteristics of the two diseases.

One finding of the study was that the number of CRS cases was projected to be low in many countries, particularly the smaller ones. This low incidence is partly because the average age of rubella infection is young and is less likely to affect pregnant women and lead to cases of CRS. However, the age of infection is likely to increase with more private sector use of rubella vaccine, leading to higher incidence of CRS.

This study findings are similar to other studies that evaluated the cost-effectiveness of measles elimination. Levin *et al* ^29^ and Babigumira *et al*^34^ both found that measles elimination was cost-effective in comparison to achieving 95% and 98% measles mortality reduction, compared with the baseline scenario of 90% measles-associated mortality reduction global goal. Acharya *et al* ^29^ also found that measles elimination would be cost-effective in Latin American countries but did not compare different service delivery strategies for achieving this outcome. Our study differed from previous ones since it utilises multiple dynamic models for comparison and includes a larger number of countries in the analysis.

The study had some limitations. First, our analysis only considered increases in coverage needed to bring measles incidence below an ‘elimination threshold’ of five cases per 1 000 000. Further activities are needed to completely break local chains of transmission and reach elimination, which will increase both the benefits and the costs of vaccination. These include outbreak response to localised outbreaks, enhanced surveillance, regional campaigns and health system improvements. The cost of outbreak response activities was omitted due to lack of information on costs and frequency (and because the benefit of these activities was also not captured by the transmission models). Second, the study was limited by being informed by only a few studies on the costs of increasing coverage and enhanced surveillance in low-income and middle-income countries. Third, the economic analyses made several assumptions on costs of surveillance, vaccination and treatment due to limited availability of these data. Fourth, the sensitivity analyses were based on arbitrary assumptions (uniform distribution with ±20% of the central estimate) and only varied case-fatality risks and economic parameters, due to lack of data to inform data-driven uncertainty distributions. Fourth, the uniform distribution was used since many of the parameters included are composites of inputs that may each be varying in different ways. In addition, many of the inputs we used were sourced from other models, which in turn have their own multiple sources of uncertainty. Fifth, the analysis was conducted before COVID so the scenarios modelled and health impacts do not account for COVID-19 pandemic disruptions. Sixth, it was assumed that countries would stay in their same World Bank income groups over the years; thus, the costs estimated over 30 years may be a lower bound.

In the future, additional economic analyses will be needed to estimate variability in costs within and between countries, and to clarify the investment required to maintain rapid outbreak response and high-quality surveillance efforts until the cases of measles and rubella reach the elimination threshold level. Further health and economic modelling will help inform strategies for the final stages of global measles and rubella elimination efforts. In the short term, the present analysis demonstrates that intensifying such efforts represents better value for money than allowing flat coverage trends to continue.

## Supplementary material

10.1136/bmjgh-2022-011526 online supplemental file 1

## Data Availability

Data are available upon reasonable request.
