# Supplementary material for: Cost-effectiveness of measles and rubella elimination in low-income and middle-income countries
Source: BMJ Glob Health. 2023 Jul 10;8(7):e011526. doi: 10.1136/bmjgh-2022-011526 (PMC10335502; doi:10.1136/bmjgh-2022-011526)
Supplement: online supplemental file 1 [file bmjgh-2022-011526supp001.pdf]

Supplement

1. Modeling Scenario Assumptions

The four vaccination scenarios have the following characteristics:

- Base Case –
  - Constant, inflation-adjusted investments in national programmes
  - All countries remain at base year coverage for the first dose of measles containing vaccine (MCV))
  - All countries remain at base year MCV2 coverage and no new MCV2 introductions beyond 2018
  - All countries remain at base year RCV coverage and no new RCV introductions beyond 2018
  - SIAs up to 2018 based on historical events and based on the current WHO rule of thumb after 2018
- Continuing Trends –
  - Limited set of improvements in national programmes
  - Historical MCV1 coverage used to fit natural log function to ramp up coverage to 99%
  - Introductions of MCV2 and RCV continue as projected by current country commitments
  - SIA frequency based on historical campaigns with cessation in countries with > 90% MCV1 & 2, RCV has been introduced, at least 5 years post-MCV2 introduction, and 2-year accumulated susceptible population less than the size of a birth cohort

- Constant Improvement –
  - Additional limited set of improvements in national programmes
  - All GAVI supported countries with MCV1 coverage <95% improve coverage from base year by 1%/year up to 95%
  - All other countries with MCV1 coverage <90% improve coverage from base year by 1%/year up to 90%
  - SIAs continue as scheduled up to 2020; after 2020 countries with MCV2 coverage <90% hold SIAs every 3 years
  - All countries introduce RCV by 2020

- Intensified Investment –
  - Increased coverage and more frequent SIAs to reach the minimum optimistic time to eradication
  - MCV1 coverage ramped up at 4.4% compounded rate up to 99% for countries not reaching 95% by 2016
  - Country-specific MCV2 introduction at forecasted coverage levels
  - Country-specific introduction of RCV during 2018-2024
  - SIA frequency based on accrual of susceptibles at 75% of size of birth cohort; cessation in countries with RCV introduction, at least 5 years post-MCV2 introduction, and MCV coverage high enough to prevent accumulation of susceptibles at 75% of birth cohort within 8 years after previous SIA

Supplemental Table 1. List of 93 Countries Included in Analysis

Afghanistan	Sri Lanka
Angola	Lesotho
Albania	Morocco
Armenia	Republic of Moldova
Azerbaijan	Madagascar
Burundi	Mali
Benin	Myanmar
Burkina Faso	Mongolia
Bangladesh	Mozambique
Bosnia and Herzegovina	Mauritania
Belize	Malawi
Bolivia	Nepal
Bhutan	Nicaragua
Cambodia	Niger
Cameroon	Nigeria
Cape Verde	Pakistan
Central African Republic	Philippines
Chad	Papua New Guinea
China	Paraguay
Comoros	Rwanda
Congo	Samoa
Cote d'Ivoire	Sri Lanka
Cuba	Sudan
Democratic People's Republic of Korea	Senegal
Democratic Republic of Congo	Solomon Islands
Djibouti	Sierra Leone
Egypt	El Salvador
Eritrea	Somalia
Ethiopia	South Sudan
Fiji	Sao Tome and Principe
Micronesia, Federated States of	Eswatini
Georgia	Syrian Arab Republic
Ghana	Togo
Guinea	Tajikistan
Gambia	Turkmenistan
Guinea-Bissau	Timor-Leste
Guatemala	Tonga
Guyana	Tunisia
Honduras	United Republic of Tanzania
Haiti	Uganda
Indonesia	Ukraine

India	Uzbekistan
Iraq	Vietnam
Kenya	Vanuatu
Kyrgyzstan	Samoa
Lao People's Democratic Republic	Yemen
Liberia	Zambia
	Zimbabwe

Supplemental Table 2. Assumptions for Measles/Rubella Routine Immunization Cost per Dose, Average Cost of Delivering a Vaccine Dose at Different Coverage Levels, and Treatment Cost (2018 USD). All figures show economic costs.

Income Group (n = no. studies)	Delivery Cost	Vaccine Price (MR)	Total	Cost per Measles-Only Vaccine	Incremental Procurement Cost per Dose of Adding Rubella Vaccine	Source
**Routine Vaccine Delivery Costs**						
Low (n=2)	$1.62	$0.66	$2.76	$2.43	$0.33	14, 15
Lower-middle (n=2)	$1.70	$2.25	$3.95	$2.82	$1.13	16, 17
Upper-middle (n=2)	$2.08	$2.25	$4.33	$3.20	$1.13	18, 19
**Campaign Costs**						
Low			$1.38	$1.05	$0.33	26,27
Lower-middle	$0.72	$0.66	$1.38	$1.05	$0.33*****	26,27
Upper-middle	$2.32	0.66	$2.98	$2.65	$0.33*****	26,27
**Marginal Cost of Delivering a Vaccine Dose at Different Coverage Levels**						
Income Group	<60%	60-69%	70-79%	80-89%	90%+	
Low (n=1)	$1.62	$1.72	$1.81	$2.06	$2.56	22
Lower-Middle (n=3)	$1.70	$1.79	$1.88	$2.13	$2.63	22
Upper-Middle (n=2)	$2.08	$2.18	$2.27	$2.52	$3.02	22
**Treatment Costs for Measles and CRS**						
**Income Group**	**Direct Cost per Case of Treatment**					
**Measles**						
Low and Lower-middle	$20.34 *					
Upper-middle	$423.12**					
**Rubella CRS**						
Low	$1,000***					
Lower-middle and Upper-middle	$6,864****					
**Additional Assumptions**						
Cost discounting rate	3%					14

Case-fatality risk (Multiplicative factor for DALYs)	1.0	0.8-1.2

Source: UNICEF Supply Division (www.unicef.org/supply/vaccines-pricing-datewww.unicef.org/supply/vaccines-pricing-date) [20] and PAHO Revolving Fund https://www.paho.org/en/documents/paho-revolving-fund-vaccine-prices-2018 [21]. *Studies in Bangladesh [27], Ethiopia [29, 30] , Tajikistan [27] and Uganda [27]; ** Based on studies in Colombia [28], Brazil [28] and Romania [5]; ***Assumption on cost of CRS treatment for low-income countries made due to lack of published data; ****Based on study in Romania [5] *****Based on 10 dose vial
n= # studies

Supplemental Figure 1. Number of vaccine doses administered 2018-2047 in 93 low- and middle-income countries by scenario, for measles and rubella

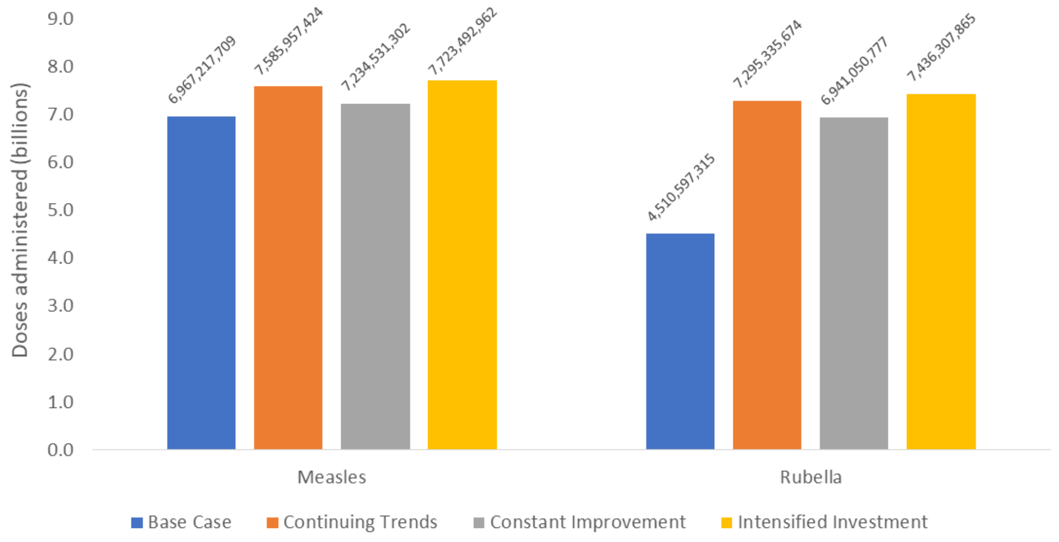

Supplemental Table 3. Cost per Measles DALY Averted for LSHTM model, (2018 USD)

Scenario	Comparator	Net Cost (billions)	DALYs averted	Cost per DALY averted
Continuing Trends	Base Case	-$3.4	379	Dominant
Continuing Trends	Constant Improvement	$590	1	$543
Intensified Investment	Continuing Trends	-$2.4	250	Dominant
Intensified Investment	Constant Improvement	-$3.5	251	Dominant

Supplemental Table 4. Incremental Cost per Rubella DALY averted for PHE model, (2018 USD)

Scenario	Comparator	Incremental Cost (millions)	DALYs averted (millions)	Cost per DALY averted
Constant Improvement	Base Case	$285	30	$10
Continuing Trends	Base Case	$644	31	$21
Intensified Investment	Base Case	$719	32	$23
Constant Improvement	Continuing Trends	$358	0.9	$406
Intensified Investment	Continuing Trends	$75	0.1	$97
Intensified Investment	Constant Improvement	$488	1.7	$294

Supplemental Figure 2. Comparisons of ICERs for PHE Rubella Model

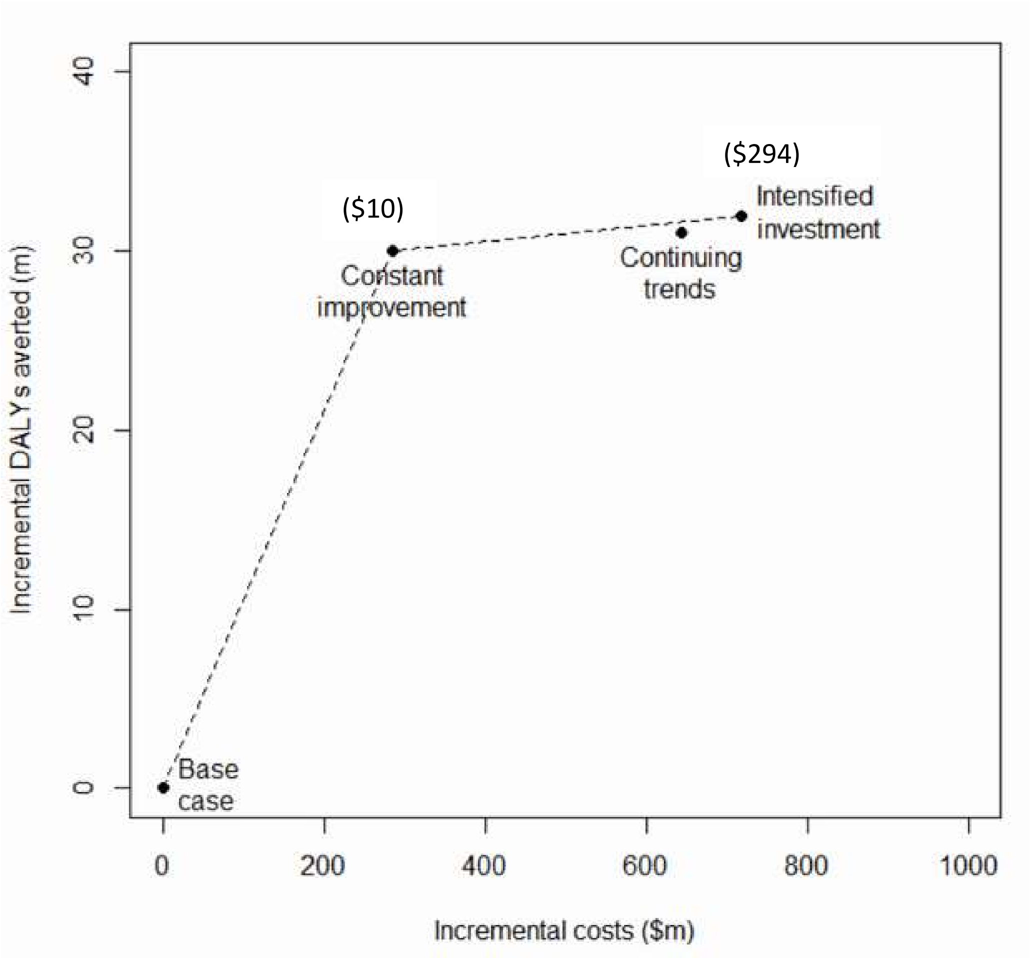

Supplemental Figure 2 shows that the Continuing Trends is extendedly dominated by a combination of Constant Improvement and Intensified Investment scenarios. Then the incremental cost effectiveness ratio of Intensified Investment compared to Constant Improvement is $294 per DALY averted. If we use the willingness to pay of $498, then Intensified Investment scenario is more cost-effective than the other scenarios.
